# Biofluid metabotyping of occupationally exposed subjects to air pollution demonstrates high oxidative stress and deregulated amino acid metabolism

**DOI:** 10.1038/srep35972

**Published:** 2016-10-21

**Authors:** Surya Narayan Pradhan, Aleena Das, Ramovatar Meena, Ranjan Kumar Nanda, Paulraj Rajamani

**Affiliations:** 1 School of Environmental Sciences, Jawaharlal Nehru University, New Delhi, India; 2 School of Life Sciences, Sambalpur University, Sambalpur, India; 3 Translational Health Group, International Centre for Genetic Engineering and Biotechnology (ICGEB), New Delhi, India

## Abstract

Occupational exposure to air pollution induces oxidative stress and prolonged exposure increases susceptibility to cardiovascular and respiratory diseases in several working groups. Biofluid of these subjects may reflect perturbed metabolic phenotypes. In this study we carried out a comparative molecular profiling study using parallel biofluids collected from subjects (n = 85) belonging to auto rickshaw drivers (ARD), traffic cops (TC) and office workers (OW). Higher levels of oxidative stress and inflammation markers in serum of ARD subjects were observed as compared to OW and TC. Uni and multivariate analyses of metabolites identified in urine by ^1^H NMR revealed 11 deregulated molecules in ARD subjects and involved in phenylalanine, histidine, arginine and proline metabolism. Despite contribution of confounding factors like exposure period, dietary factors including smoking and alcohol status, our results demonstrate existence of exposure specific metabotypes in biofluids of ARD, OW and TC groups. Monitoring serum oxidative stress and inflammation markers and urine metabolites by NMR may be useful to characterize perturbed metabolic phenotypes in populations exposed to urban traffic air pollution.

Occupational health hazards are the key causalities for multiple disease conditions in various vulnerable populations in urban settings. Long term exposure to vehicular exhausts such as ultrafine particulate matter (UFP) with diameter of 10/2.5 micrometers (PM_10/2.5_), heavy metals, exhaust gases (NOx, SO_2_, CO) and polyaromatic hydrocarbons (PAH) are associated with higher risk of developing asthma, cardiovascular, infectious diseases and cancer in professional drivers^1^,^2^. A recent report indicated outdoor air pollution may double premature mortality by 2050^3^ and it poses a grave concern. Exposure to traffic pollutants contributes higher pulmonary deposition of UFP and increased entry to circulation triggers higher reactive oxygen species (ROS) production in various pulmonary and extrapulmonary vascular endothelium^4^. Impaired oxygen saturation in lungs may perturb metabotypes of circulatory and excretory biofluids of these vulnerable populations.

Continuous exposure to particulate urban air pollution in metro cities has been an increasing health concern to general public and vulnerable populations. Local truck drivers spending more time in polluted areas such as streets with heavy traffic and construction sites showed higher exposure to dust (0.3 mg/m^3^)^5^. Apte *et al*. reported that particle concentrations measured in auto rikshaws in Delhi were much higher (geometric mean for w60 trip-averaged concentrations: 190 μg/m^3^ PM_2.5_, 42 μg/m^3^ black carbon mass: BC, 280 × 10^3^ particles number cm^3^: PN) than other megacities^6^.

High serum oxidative stress and inflammation markers levels in serum of occupationally exposed taxi and bus drivers have been reported^7^,^8^,^9^. Auto rickshaw provides a popular, easy, cost-effective solution to reach the last miles of destination in urban and rural populations of underdeveloped and developing countries belonging to Asia, Africa, South America and Europe. Studies revealed that subjects travelling in auto rickshaws get exposed to higher level of traffic pollution than ambient levels (by 1.5 × for PM_2.5_, 3.6 × for BC, and 8.4 × for PN)^6^. Very limited study has been attempted to metabotype biofluids of auto rickshaw drivers (ARD) to investigate their perturbed health status^10^. These subjects spends ~8 hrs/duty day in traffic and study on the effect of air pollution in ARD could also aid in understanding its impact in other vulnerable groups like bikers, motorists, drivers using paddle or electric powered tricycles to ferry people or material and open car. Monitoring known oxidative stress and inflammation markers in serum and urine metabolic profiling by ^1^H NMR provides useful information to decipher altered metabolic phenotypes in subjects exposed to different pollutions^11^,^12^,^13^.

Therefore, in this study, we monitored serum oxidative stress and immunological markers and untargeted urine metabolic profiling of ARD subjects as case and office workers: OW and traffic cops: TC as controls, to identify important deregulated metabolites. Molecular pathways that are significantly altered in ARD with comparison to OW were explored and compared with TC groups. To best of our knowledge, this research is the first to combine oxidative stress and metabolomics to report perturbed molecules and pathways in parallel biofluids of high vulnerable populations like ARD.

## Methods

### Ethical statement

Institutional ethics review board of Jawaharlal Nehru University approved this study (IERB-JNU-12:PhD/02.05.2012). Subject recruitment, sample collection and analysis were employed in accordance with the relevant guidelines and regulations. All study subjects provided written informed consent to participate in this study.

### Study population and exposure rate calculation

**Figure 1:**
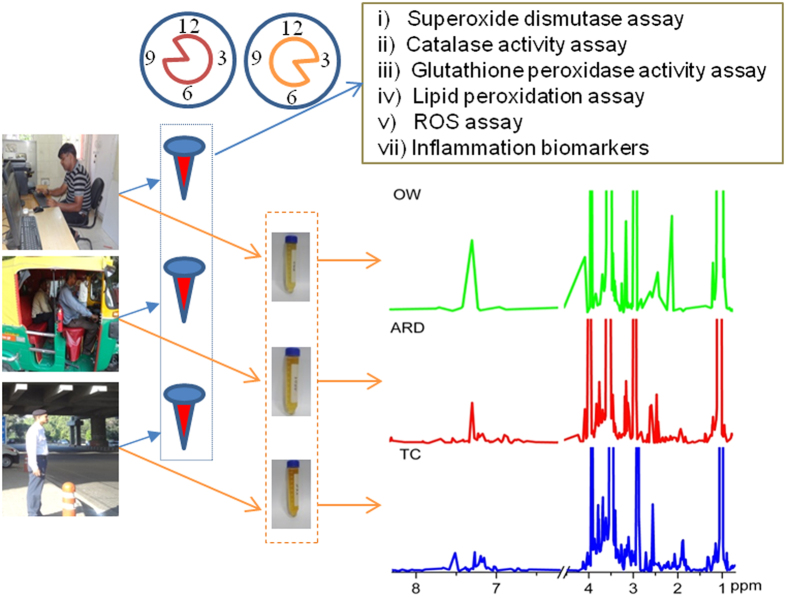
Schematic workflow used in this study to explore existence of oxidative, inflammation and metabolite specific phenotypes in biofluids of three study groups like office worker (OW), auto rickshaw driver (ARD) and traffic cop (TC). Blood (~5 mL) samples were collected by 9–11 am and mid stream on-spot urine samples (~40 mL) were collected between 3–5 pm from all study subjects. Serum samples were used for quantifying oxidative and inflammatory stress markers states of these study groups. Representative 500 MHz ^1^H NMR spectra of urine samples collected from OW, ARD, and TC subjects are presented. ROS: reactive oxygen species.

A total of 85 subjects (all male, mean age 33 and range 25–40 in years) belonging to three groups (Office Workers: OW, n = 23; Auto Rickshaw Driver: ARD, n = 29; and Traffic cop: TC, n = 33) were recruited from specific locations (Indira Gandhi International airport: 77.099819°E: 28.556047°N; Rama Krishna puram: 77.180699°E: 28.560547°N; Khelgaon: 77.213070°E: 28.553112°N; Punjabi bagh: 77.142069°E: 28.672904°N and Arjun nagar: 77.295143°E: 28.655646°N) in Delhi between August 2013 to May 2014. Subjects above 18 years old, and not taking any kind of medication two weeks prior to the sample collection, were included for this molecular profiling study and steps followed are schematically presented in Fig. 1. The demographic details including self reported health status of all study subjects were collected in a questionnaire (Table 1). Monthly temperature and humidity information were collected from the Indian Meteorological Department. Annual (1^st^ Aug 2013-31^st^ July 2014) PM_10_ and PM _2.5_ data from one of the Delhi station (i.e. Indira Gandhi International airport) was collected from Central Pollution Control Board (CPCB), Ministry of Environment and Forests, Government of India (Supplementary Table S1 and S2). Adjusted air concentration of all study groups were calculated using Equation 1.


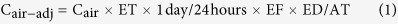


C_air_: Concentration of contaminant (PM 2.5/10) in air (mg/m^3^) from CPCB; ET: Exposure time (hours/day); EF: Exposure frequency (days/year); ED: Exposure duration (years); AT: averaging time (days).

For different sectors (including OW and TC) on average 270 days are working days so EP was fixed at 270 days. All study groups spend different exposure time (OG: 2 hours; ARD: 8 hours; and TC: 6 hours) per day on road. Average body weight of the study population was used for calculating the adjusted air concentration (C_air-adj_).

### Lung function assessment

Spirometric parameters such as forced expiratory volume in 1 sec (FEV_1_) and forced vital capacity (FVC) of study subjects were monitored using a spirometer (Minispir MIR, Italy) following the standardized methodology recommended by American Thoracic Society^14^. A trained person carried out respiratory measurements and a single instrument was used to complete data acquisition. The tests were performed in a sitting position of the subject, nose clips in place and at least five spirometric tests to get at least three reproducible and acceptable maneuvers, to calculate the lung function parameters.

### Biofluid collection and storage

From each study subjects, single blood sample (~5 ml) was drawn from the median cubital vein by a paramedical staff and collected in 15 ml sterilized falcon tubes during 9–11 am. Blood samples were transported from collection site to research laboratory in ambient temperature. Serum extraction was carried out in the laboratory by incubating blood samples at 37 °C for 2 hours and centrifuged at 2,152 × *g* for 10 min at 4 °C to collect supernatant. Aliquot of serum samples (500 μl) were stored at −80 °C for further analysis. Similarly, single random mid stream urine sample was collected in pre-chilled 50 mL falcon tubes from all study subjects between 3–5 pm i.e. just after completion of approximate 6–8 hours of exposure to ambient air pollutants. Urine samples were stored and transported in ice from the collection site to research laboratory for preprocessing. After centrifuging at 8,000 × *g* for 10 min at 4 °C, inhibitors (33 μL of 100 mM sodium azide, 500 μL of 2% phenylmethylsulfonyl fluoride and 100 μL of 1 mM leupeptin for 50 mL urine) were added to supernatant before storing at −80 °C until further analysis. Steps from sample collection to processing were completed within 5–6 hr and code was assigned on the same day. No more than two freeze thaw cycles before data acquisition was allowed for each sample.

### Lipid Peroxidation Assay

Serum lipid peroxidation was estimated by measuring thiobarbituric acid reactive substances (TBARS) and expressed in terms of malonyl dialdehyde (MDA) formed per mg protein. Briefly, 40 μL of serum sample mixed with 1.6 mL of 10 mM Tris-KCl (0.15 M KCl and pH 7.4) buffer, 0.5 mL of 30% trichloro acetic acid (TCA) and 0.5 mL thiobarbuturic acid (TBA) in falcon tubes and covered with aluminium foil. Reaction mixtures were incubated in water bath maintained at 80 °C for 45 min and subsequently cooled in ice and centrifuged (5,000 × *g*) at ambient temperature for 10 min in a tabletop centrifuge. The absorbance of the clear supernatant was measured against reference blank of Milli-Q water at 531.8 nm in a spectrophotometer (CARY 100 Bio/Varian, Australia). Total protein content of serum samples was estimated following Bradford method and bovine serum albumin was used as standard^15^.

### Glutathione peroxidase activity assay

Undiluted serum sample (25 μL) and 100 μL of 2 mM hydrogen peroxide was added to freshly prepared reaction mixture consisting of 755 μL of 0.1 M sodium phosphate buffer (pH 7.0), 100 μL of 2 mM nicotinamide adenine dinucleotide phosphate (NADPH), 10 μL glutathione reductase (2 μL in 500 μL buffer) and 10 μL reduced glutathione (9.22 mg/mL) kept in cuvette of 1 mL capacity. Oxidation of NADPH was followed for 3 min, and absorbance at 340 nm was recorded every 1 min. One unit of enzyme activity was reported as 1 μmol NADPH oxidized/min assuming 6.22 × 10^3^ to be the molar absorbency of NADPH at 340 nm. The specific activity of Glutathione peroxidase (GPx) was expressed as μmoles of NADPH oxidized/min/mg protein as explained by Mills 1959^16^.

### Catalase activity assay

Ten times diluted serum (20 μL) sample, in sodium phosphate buffer (0.1 M, pH 7.4), was added to 10 μL of Triton-X 100 (1%) and incubated at 4 °C for 30 min. Absorbance of reaction mixture with 0.98 mL of 0.03 M H_2_O_2_ in phosphate buffer, was recorded at 240 nm for 3 min at 30 sec intervals. Catalase activity was expressed as nkat per mg protein and one kat is defined as 1 mole of H_2_O_2_ consumed per second per mg protein as explained earlier^17^.

### Superoxide dismutase assay

Superoxide dismutase (SOD) activity was determined following standard method^18^. Diluted serum sample (10 μL: 10 times diluted with 0.1 M sodium phosphate buffer, pH 7.4) and 10 μL Triton-X 100 (1%) was incubated at 4 °C for 30 min. Processed sample was added to 1 mL of assay mixture (1 mL of 0.05 M sodium phosphate buffer pH 8.0, 0.01 M EDTA and 0.27 mM pyrogallol) and absorbance was scanned for 5 min at 420 nm in 1 min interval using a spectrophotometer. Enzyme activity was expressed in U/mg protein, where 1U is the amount of enzyme required to bring about 50% inhibition of the auto oxidation of pyrogallol.

### ROS assay

Total serum reactive oxygen species (ROS) was estimated following a method reported by Hayashi *et al*.^19^. Briefly, 5 μL of either hydrogen peroxide (for generating a calibration curve) or sample was added to 140 μL of 0.1 M sodium acetate buffer (pH 4.8) in each well of a 96-well microtiter plate and incubated for 5 min at 37 °C. Fixed volume (100 μL) of freshly prepared reaction mixture by mixing R1 (100 μg/mL of N, N-diethyl-para-phenylendiamine in 0.1 M sodium acetate buffer at pH 4.8) and R2 (4.37 μM of ferrous sulfate in 0.1 M sodium acetate buffer pH 4.8) in a ratio of 1:25 was added to each well. After incubating for 1 min at 37 °C, absorbance was measured continuously for 360 sec at 30 sec intervals using a plate reader at 505 nm. Serum ROS level was calculated using calibration curve and expressed as hydrogen peroxide equivalent (1 unit = 1.0 mg H_2_O_2_/L).

### Inflammation biomarkers

Serum level of interleukins (IL-6, IL-1α and TNF-α) were measured using commercial enzyme-linked immunosorbent assay kits (IL-6/ IL-1α/ TNF- α human EIA Kits 501030/583301/589201 respectively from Cayman Chemical, USA) following manufacturer’s instructions. Measurements were done in duplicates with ten times diluted serum sample and inter-assay variability was found to be within 10% of each other. According to the manufacturer’s product insert, the minimum detectable concentration of IL-6, IL-1α and TNF- α were 7.8 pg/mL, 3.9 pg/mL and 7.8 pg/mL, respectively.

### ^1^H NMR Data Acquisition of Urine Samples

Coded urine samples were randomized using RAND function in Microsoft Office Excel 2007 to remove biasness and 6–8 samples per day were processed as a single batch. Urine samples were thawed in ice, centrifuged at 4 °C for 30 min and subsequently kept at ambient temperature for 30 min. Urine sample (540 μL) and 60 μL of reaction mixture (1.5 mM of 3-(Trimethylsilyl)propionic-2,2,3,3-d_4_ acid sodium salt (TSP) in D_2_O, Sigma-Aldrich) was transferred to 5 mm NMR tubes (Wilmad-Lab Glass, Sigma, USA) for NMR measurements. ^1^H-NMR data acquisition of all study samples was completed within 34 days. ^1^H NMR spectra were acquired using a Bruker Advance III spectrometer (BrukerBiospin GmbH, Germany) equipped with 5 mm PABBO-BB probe head, operating at the field strength of 500.13 MHz. Temperature calibration was performed using methanol-d_4_ (D-99.6%). All NMR spectra were phased and baseline-corrected. Chemical shifts were referenced to the TSP signal. NMR spectral data were processed using Topspin 3.1 (Bruker Corporation, Rheinstetten, Germany). All ^1^H NMR spectra with water suppression by excitation sculpting using the zgesgp pulse sequence were measured with 8 scans with a relaxation delay of 2 sec ref. 20. For each sample free induction decays (FIDs) were collected with a spectral width of 10,000 Hz and acquisition time of 2.7 sec (t_1_max). Data from each sample was accumulated into 32 k data points within a spectral width of 12 ppm. Spectral intensities were scaled and reduced to integrating regions of equal width (0.01 ppm) within the spectral region of 8.5–0.2 ppm. To remove the effects of suppression of water resonance and variation in the urea signal caused by partial cross-solvent saturation due to solvent- exchanging, proton region between 4.50 and 6.20 ppm was set to zero integral in the spectrum.

### Data pre-treatment and statistics

Each NMR data file was manually screened for data consistency. Peak areas of each molecular feature were added from every study sample. Peak lists of the 52 data files (OW: n = 23 and ARD: n = 29) were uploaded in recommended zipped format to a web based software MetaboAnalyst 2.0 for statistical analysis^21^. Resultant raw data matrix was used for uni-variate analysis and total area normalized data matrix was used for multivariate analysis. Peaks present in more than 50% of all data of each study groups were included. Missing value imputation was carried out by including a minimum value of 98.72 from the complete data matrix. Generalized logarithm transformation, and pareto scaling was carried out before undertaking further multivariate analyses.

Principal component analysis (PCA) using all qualified molecular features were carried out to explore the grouping and outliers, if any. Classification model using partial least square discriminate analysis (PLS-DA) was built using the entire matrix. A combination of uni-variate (fold change: FC = ARD/OW > 2.0 at Wilcoxon Mann Whitney p-value < 0.05) and multivariate analysis (variable importance in projection: VIP score > 1.4 of PLS-DA plot) was used to identify important molecules that could be useful to explain the inter class variations between ARD and OW. Peak areas of these selected metabolite features were extracted from all three study groups (TC: n = 33; OW: n = 23 and ARD: n = 29) were used for PLS-DA analysis to explore clustering patterns.

### Pathway Enrichment Analysis

These important molecules were included for pathway enrichment analysis using MetaboAnalyst 2.0 to identify the deregulated metabolic pathways in ARD^21^.

### Statistical Analysis

The statistical significance of the difference between the means of the three groups was assessed using the two-sample t- test or the nonparametric analogue Wilcoxon Man Whitney test. A p-value < 0.05 (confidence level 95%) was considered statistically significant. The box plot representation was used to visualize the variation in the levels of integrated analytes between case and control groups and prepared using ORIGIN 7.5 (OriginLab Corporation USA). All data were presented in mean ± SD.

## Results

### Subject and sampling sites details

Table 1 shows the demographic details of all study subjects belonging to three study groups at recruitment and sample collection (n = 85; OW:23, ARD:29, and TC:33). Of the total 85 subjects recruited in this study, 100% were male, mean age 33 years (range 25 to 40 years) and the mean body mass index (BMI) was 23.8 kg/m^2^ (s.d.: 1.16). Study subjects reported skin disease (16%), high blood pressure (9%), cough (13%), dust allergy (27%), smoking (44%), alcohol use (43%) and eye irritation/burning (47%). A small fraction (4–7%) of these subjects reported to have symptoms of tuberculosis, asthma and respiratory obstructions. Average ambient temperature and relative humidity values at the clinical site ranged from 22.8 °C to 37.64 °C and from 33% to 73% respectively.

### Adjusted air concentration of PM 2.5 and 10

As per the available data, annual PM_2.5_ and PM_10_ air exposure concentration in ARD was highest (0.063 and 0.079 mg/m^3^), lowest in OW (0.015 and 0.019 mg/m^3^) and intermediate in TC (0.047 and 0.059 mg/m^3^).

### Respiratory outcomes of ARD, OW and TC

We did not observe strong relationships in their respiratory outcomes between ARD, OW and TC groups. Although sample size is low, we did not find active smoking habit influencing the respiratory conditions of ARD, OW and TC (Wilcoxon-Mann-Whitney t test, p > 0.1; Supplementary Fig. S1). The detailed results of FEV_1_, FVC and FEV_1_/FVC (%) between study groups are presented in Table 1 (Supplementary Fig. S1).

### Oxidative stress markers and ROS

**Figure 2:**
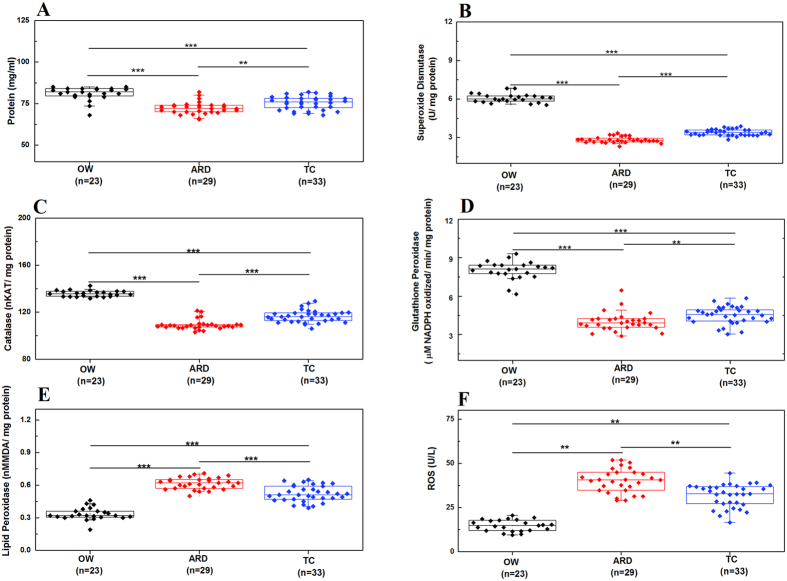
High oxidative stress and reactive oxygen species (ROS) levels are observed in auto rickshaw driver (ARD) subjects with respect to office worker (OW) and traffic cop (TC). Levels of Protein (**A**), Lipid Peroxidase (**B**), Catalase (**C**), Glutathione Peroxidase (**D**), Superoxide Dismutase (**E**), and Reactive Oxygen Species (ROS) (**F**) in study groups are presented in box and whisker plots (**^,^***p value < 0.01, and < 0.005, respectively, of Wilcoxon-Mann–Whitney test). Black diamond shows OW subjects, red for ARD and blue diamond’s for TC. Number of subjects used in this study is presented in parenthesis.

Total serum protein was found to be lower in ARD than OW and TC groups (Fig. 2A). With respect to OW decreased activity of SOD and catalase were observed in serum of both ARD and TC subjects (Fig. 2B,C). We also observed significant differences in serum SOD activity between ARD (2.82 ± 0.23 U/mg protein) and TC (3.39 ± 0.24 U/mg protein). Similar trend was also observed in non-enzymatic glutathione peroxidase activity (Fig. 2D). Higher lipid peroxidase activity was observed in ARD (0.61 ± 0.05 nM MDA/mg protein) subjects with respect to TC (0.52 ± 0.07 nM MDA/mg protein) and OW (0.33 ± 0.06 nM MDA/mg protein) (Fig. 2E). Minimum lipid peroxidase activity was observed in subjects belonging to OW groups. Serum ROS were found to be higher in subjects belonging to ARD (40.13 ± 6.76 U/L) and TC (31.7 ± 44.77 U/L) than OW (14.69 ± 3.27 U/L) group (Fig. 2F). Additionally, ROS concentration was found to be significantly higher in ARD subjects then TC groups.

### Inflammation markers

**Figure 3:**
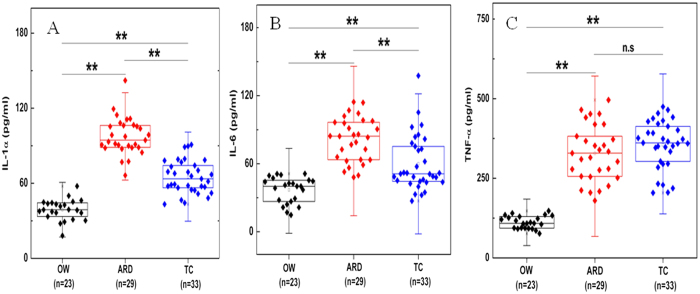
Significantly higher levels of inflammation markers were found in auto rickshaw driver (ARD) subjects with comparison to office worker (OW) groups. Concentrations of IL-1α (**A**), IL-6 (**B**) and TNF-α (**C**) in OW, ARD and traffic cops: TC subjects are presented in box and whisker plot (**p value < 0.01 of Wilcoxon-Mann–Whitney test and n.s.: not significant at 95% confidence). Black diamond shows OW subjects, red for ARD and blue diamond’s for TC. Number of subjects used in this study is presented in parenthesis.

Higher concentration of inflammation markers such as IL-1α and IL-6 were observed in ARD (81.3 ± 19.34 pg/mL and 97.55 ± 14.83 pg/mL) subjects, lower in TC (60.07 ± 24.28 pg/mL, and 64.95 ± 13.05 pg/mL) and minimum in OW (36.89 ± 11.49 pg/mL, and 38.86 ± 8.35 pg/mL) groups (Fig. 3A,B). TNF-α concentration was the highest in TC (355.11 ± 76.16 pg/mL) followed by ARD (328.64 ± 87.68 pg/mL) and minimum in OW (111.72 ± 19.76 pg/mL) subjects (Fig. 3C). Statistically significant variations in IL-6 and IL-1α levels were observed between all three study groups whereas not much variation in abundance of TNF-α between ARD and TC was observed.

### Comparative Urine Metabolite Profiling of ARD and OW subjects to Identify Important Metabolites

**Figure 4:**
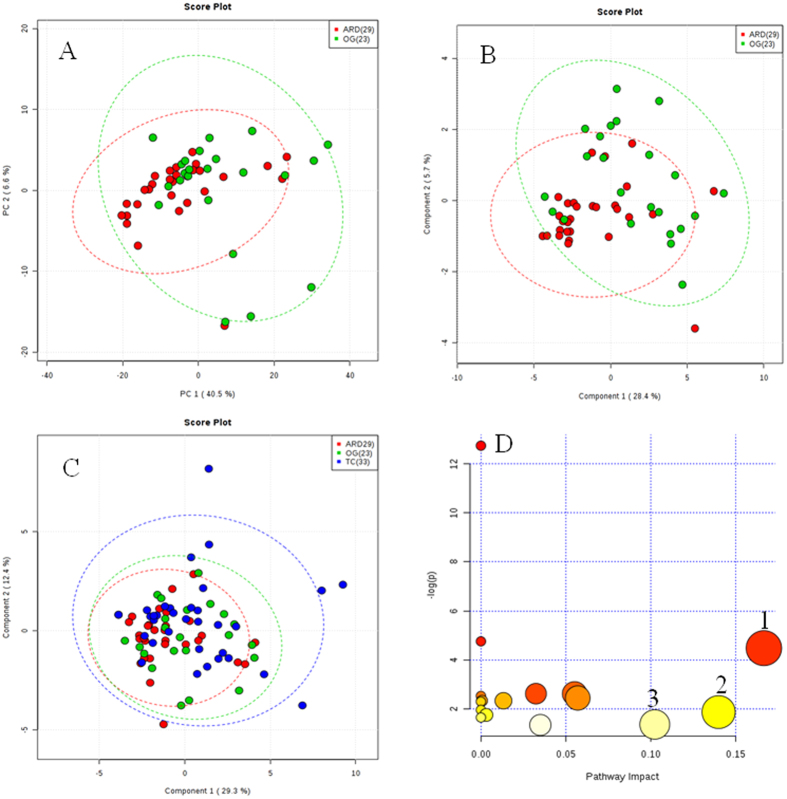
Urine metabolic phenotype of office worker (OW), auto rickshaw driver (ARD) and traffic cop (TC) show variation. (**A**) Score plot generated from principal component analysis (PCA) using 15 peak groups from OG and ARD to identify any outliers; (**B**) Score plot generated from partial least-squares discriminate analysis (PLS-DA) using 11 peak groups from OW and ARD; (**C**) Overview of altered metabolic pathways as reflected from the deregulated molecules in urine of ARD subjects compared with OW. Bubble area is proportional to the impact of each pathway with color denoting the significance from highest in red to lowest in white. (1) Phenylalanine metabolism, (2) Histidine metabolism and (3) Arginine and proline metabolism and (**D**) Score plot generated from revised PLS-DA adding 11 peaks from TC. Green dots show OW, red for ARD and blue dots for TC. Number of subjects used in this study is presented in parenthesis.

After careful manual check and selecting variables, meta data from two study groups (ARD:29 and OW:23) with 135 peak groups showed high degree of overlapping clusters (Fig. 4A). A partial least square discriminate analysis (PLS-DA) model built using this data matrix, we observed large portion of subjects grouped in two clusters (Fig. 4B). A list of 15 peak groups have VIP score of >1.40. From Mann-Whitney-test we observed at least 65 molecules showed fold change (FC: ARD/OW > 2.0 at p value < 0.05). Moreover, 11 molecules qualified both these parameters and identified from Human Metabolome Database (HMDB) as 2-hydroxy valerate, kynurenine, proline, histidine, cis-aconitic acid, benzoic acid, p-cresol sulfate, 4-amino hippuric acid, phenylalanine, isoleucine and alanine. This important set of molecules could differentiate ARD from OW with high accuracy (Table 2).

### Metabolic Pathway analysis

We observed 3 metabolic pathways (phenylalanine, histidine, arginine and proline metabolism) to be significantly getting deregulated in ARD subjects with respect to OW (pathway impact threshold >0.10) (Fig. 4C). These metabolic pathways may explain perturbed metabolic phenotypes in these subject groups exposed to urban traffic air pollution.

### Abundance of Important Metabolites in a Control group (TC)

Comparing the abundance of 11 important deregulated metabolites between ARD and TC groups showed statistically insignificant variation and indicates high similarity between both these groups (Supplementary Fig. S2). The PLS-DA model built using the important molecular features identified from ARDs and OW comparison, was found to misclassify the TCs groups (n = 33) explaining the importance of these metabolites and its correlation to exposure to traffic air pollution (Fig. 4D). Variations in abundance of individual molecules from all three study groups are presented (Supplementary Fig. S2). More importantly, 3 important molecules (2-hydroxy valerate, kynurenine and alanine) were found to be significantly altered in both ARD and TC groups with respect to OW. Rest eight molecules showed insignificant variation between OW and TC groups.

## Discussion

In this study, we attempted to explore whether serum oxidative stress markers and urinary metabolites of subjects exposed to traffic air pollution irrespective of their smoking status could be useful to understand their perturbed metabolic phenotype. Common platforms for providing method of transportation vary in developed, developing and underdeveloped countries from Asia, Africa, South America and Europe. Auto rikshaw provides a cost effective method of transportation in resource limited settings. Like taxi and truck drivers, ARDs every working day get exposed to urban air pollution with higher concentrations of ultrafine particles and gaseous components. Control groups like OW and TC in urban settings also do get exposed to similar air pollutants but the exposure periods and intensities may be different and found to be appropriate control groups for a comparison to exposure induced metabolic alterations in ARDs. The different levels of exposure in OW, ARD and TC groups may have contributed to such oxidative stress and inflammation level variations. Epidemiological studies have shown up to 1.5 time’s elevated risk of lung cancer in subjects staying in urban or industrially polluted areas^22^. Diesel exhaust exposed occupational groups provide support for a causative role of traffic related air pollution for lung and cardiovascular related diseases^23^.

All study subjects were recruited between Aug-2013 to May-2014 to minimize effect of season specific variations as reported earlier in vulnerable groups like in bus drivers^8^. Respiratory parameters of these three study groups (OW, ARD and TC) were found to be similar. Smoking habit of ARD subjects did not show difference in respiratory parameters between OW and TC groups.

It is believed that air pollutants like diesel exhausts and UFP play a key role in oxidative stress mediated damage leading to increased morbidity and mortality and majority of them due to cardiovascular and respiratory disease and cancer^24^,^25^. Oxidative damage could result at protein, lipids and DNA levels and in this study we attempted to monitor oxidative stress marker molecules at protein and lipid levels^11^.

Proteins are major components of biological systems, and can react directly with ROS or indirectly with other oxidized products of lipids or sugars. Both PM and surface attached transition metals generate hydroxyl radicals through Fenton reactions^26^. Direct oxidation alters conformation of proteins leading to inactivate function, the cleavage of peptide bonds or in heavy oxidation leading to proteolysis. Minimum total serum protein concentration was observed in ARD groups with respect to TC and OW, and reflects shift in protein turn over. Further validation by monitoring protein carbonyl levels may provide useful information about the role of protein oxidation for such outcomes. In this study, we observed similar oxidative stress marker levels between ARD and TC subjects and lower in OW. Duration, type and strength of exposure to air pollution may contribute for such phenotypic variations.

ARDs not only showed the highest levels of oxidative stress in terms of ROS and lipid peroxidase activity but also had the lowest proportion of catalase, superoxide dismutase and glutathione peroxidase activity. Although oxidative stress might have caused due to both exo and endogenous factors, important cellular homeostasis function tries to maintain balance between ROS and antioxidants defense. This balance may have shifted in subjects occupationally exposed to traffic air pollution like the ARD and TC study groups. Relatively higher exposure to pollutants as reflected from working hours might have increased ROS production leading to oxidative stress in ARD. These observations suggest that while over exposure to environmental pollution may have the potential to induce oxidative stress, other factors may also play major role in existence of a different tier in the oxidative response of ARD and TC. Mental stress caused by the driver’s responsibility for the passenger wellbeing and safety may be one of the additional factors that cannot be measured exactly in ARDs.

Furthermore, alveolar macrophages provide first level of protection by ingesting and removing the inhaled particles from the lungs^27^. Activation of alveolar macrophages leads to release of cytokines^28^ and in our study we observed significantly higher concentrations of serum IL-1α, IL-6 and TNF- α in ARD with respect to OW. However, other than TNF- α levels, significant variation in IL-1α and IL-6 concentrations between ARD and TC groups were observed. In addition, it is interesting that the increased IL-6 levels in ARDs could be related to a shift towards a procoagulant state. These findings suggest that inflammation levels of ARDs and TCs do show significant levels of similarities with minor differences.

The overall calculated annual PM 2.5 and PM 10 exposure was found to be highest in ARD subjects. The exposure data of PM 2.5 and PM 10 were calculated, from the available data points of 127 days and 62 days respectively, from one of the station maintained by CPCB. ARD subjects get at least 4 and 1.3 times higher exposure with respect to OW and TC respectively.

The variation in levels of serum markers are noteworthy and in line with a previous studies noting significant alterations in these indices in bus drivers^8^,^29^. These observations are therefore in agreement with the hypothesis that over exposure to environmental pollutants can affect oxidative and inflammatory markers. However, since the observed differences in ARD higher than TC groups, it is interesting to correlate them with long term overall health outcome.

PCA analysis of NMR data was used to detect the inherent clustering and we did not observe any outliers within these study groups. We did observe a set of 11 important deregulated urine metabolites could differentiate ARD from OW but many of them show insignificant variation between ARD and TC groups. It is important to note that higher molecular abundance of 2-hydroxy valerate, histidine and cis-aconitic acid were observed in both ARD and TC groups with respect to OW. These three important molecules may be useful as indicators of degree of exposure of air pollution and need further investigation by including carefully selected study groups in longitudinal follow up studies.

The important deregulated amino acids play significant role in explaining increased oxidative stress in ARDs. Loss of amino acids is primarily due to oxidative metabolism and is important to maintain adequate protein and amino acid balance in body. May be protein turnover is higher in these occupationally exposed subjects and even a small increase in protein degradation or a decrease in protein synthesis rate, persisting for longer periods may cause loss of lean body mass. High concentration of propionate derived ketone bodies such as 3-hydroxy valerate may explain metabolic decompensation in ARDs as explained in methylmalonic acidemia and propionic academia^30^,^31^. Increased kynurenine reflect an important compensatory pathway for the regulation of vascular function in inflammatory conditions and relevant to endothelium derived vasodilators^32^. Potential toxic compounds like p-cresol are produced by gut microbiota and higher concentration of it is partly influenced by diet composition^33^. Cresol excretion was found to be lowered by administration of prebiotic substrate along with *Lactobacillus caseishirota* and *Bifidobacetrioum breve* to human subjects^34^. Increased cresol level in urine of ARD subjects may be due to altered gut microbiome diversity or different food habits or both.

Amide derivative of glycine and para-aminobenozoic acid leads to formation of p-aminohippuric acid (PAH) and increased levels of it in ARD may show impaired renal function^35^. Higher elimination of phenylalanine in urine of ARD may also reduce catecholamine production and that may interfere with the antioxidant repair mechanism^36^. Benzoate was one of the compounds first found to be elevated in urine of patients with intestinal bacterial over growth of various origins^37^. And bacterial catabolism of dietary polyphenols may be the predominant origin of benzoate, which is normally conjugated with glycine in the liver to form hippurate^38^. Abnormalities of urinary benzoate and hippurate may reveal clinically significant detoxification or dysbiosis issues^39^. Higher benzoate in urine of ARD indicates poor detoxification via phase II glycine conjugation. In separate studies, deregulated amino acid levels has been reported in subjects with long term environmental exposure to cadmium or arsenic^40^,^41^.

The strengths of the present study merit discussions. First, to the best of our knowledge, this is the first focused combined studies to identify effect of occupational exposures induced altered oxidative stress and metabolic phenotypes in important study groups like ARD and TC. Second, these subjects are recruited from 4 different locations to have wider opportunity to compare between multiple exposure groups. Third, by using proposed important metabolites to evaluate exposure stress, more accurate health evaluation could be made, then only measuring the occupation associations.

The present study also has some limitations. Only male subjects were used in this study to represent the current ARDs in Delhi and show 5 times higher occupational risk factors associated deaths. We measured metabolic and other physiological details to explain the oxidative stress but due to operational challenges, individual exposure monitoring to pollutants such as NOx, CO, PM or PAH and noise disturbance were not accounted for in this study. Only annual PM_2.5_ and PM_10_ data were available and used to calculate the exposure rate in all three study groups. The overall health outcomes may principally be contributed to exposure associated with their profession however other factors like socioeconomic status, tobacco smoking, dietary intake and use of different fuel at home for cooking might have partly contributed to these variations. However, we used a questionnaire to collect information about possible contributing factors and tried to adjust in analyses, but residual confounding factors may persist. Longitudinal follow up studies to monitor time dependent effect of air pollution on the oxidative stress, inflammation and change in metabolic phenotypes of ARD subjects would be interesting but several challenges including migration for better opportunity and frequent change in local address need attention.

It is interesting to note that many (>40–50%) of ARD study subjects complained of dust allergy, eye irritation and none of them used any sorts of personal protective gears. Appropriate focused intervention study to recommend protective cost effective solutions such as masks to reduce injury to lung epithelial tissue and administration of appropriate nutraceuticals or good microbiota to manage oxidative stress in these high risks groups like ARD and TC would be interesting avenues.

In this study, we report how metabotyping of serum markers and micromolecules of non-invasive biofluids like urine of occupationally exposed groups could be useful to understand deregulated pathways which may explain exposure specific variation. We monitored the oxidative stress serum markers in all three study groups and employed ^1^H NMR to carry out metabolite profiling of urine and compared the molecular profiles of ARD and TC with OW. Employing advanced statistics we identified a group of metabolites that are significantly deregulated in ARD subjects. Further comparison between ARD and TC, owing to their similar exposure, were carried out to explore whether they share comparable metabolic phenotypes or not.

## Conclusion

In this paper, we have demonstrated usefulness of monitoring oxidative stress markers and urine metabolic profiling of using ^1^H NMR as useful tools to understand perturbed metabolic phenotypes of subjects having occupational exposure to traffic air pollution. We demonstrated for the first time, the combined existence of elevated oxidative stress marker levels and altered urine metabolic profiles in ARD with respect to OW but similar to TC by a comparative metabolic profiling study. A decade long duration longitudinal follow up study or ARD group and simultaneous exposure monitoring, altered metabolic profile and clinical outcomes could be attempted in future to further validate these findings. However, this study provides sufficient evidence that identification of deregulated molecules could be useful to monitor few endogenous metabolites to monitor metabolic phenotype of high risk subjects with occupational exposure to air pollution and may facilitate further development of preventive or therapeutic care in future.

## Additional Information

**How to cite this article**: Pradhan, S. N. *et al.* Biofluid metabotyping of occupationally exposed subjects to air pollution demonstrates high oxidative stress and deregulated amino acid metabolism. *Sci. Rep.* **6**, 35972; doi: 10.1038/srep35972 (2016).

## Supplementary Material

Supplementary Information

Supplementary Table S1

Supplementary Table S2

## Figures and Tables

**Table 1:** Demographic details of all study subjects.

Subject details	Total	Auto rickshaw driver (ARD)	Office worker (OW)	Traffic cop (TC)
Number of subjects	85	29	23	33
Mean age (range) in years	33 (25–40)	32(25–38)	33 (28–40)	33 (27–39)
Gender (Male in %)	100	100	100	100
Height (Mean ± SD in cm)	168.5 ± 2.9	166.6 ± 2.7	167.2 ± 1.6	171.2 ± 1.3
Weight (Mean ± SD in kg)	67.4 ± 4.55	64.6 ± 4.9	67.9 ± 2.78	69.5 ± 3.9
BMI (Mean ± SD)	23.8 ± 1.16	23.4 ± 1.2	24.3 ± 0.72	23.7 ± 1.3
FVC (Mean ± SD in L)	2.15 ± 0.27	2.12 ± 0.26	2.15 ± 0.26	2.19 ± 0.29
FEV_1_ (Mean ± SD in L)	1.77 ± 0.14	1.75 ± 0.16	1.77 ± 0.14	1.77 ± 0.19
FEV_1_/FVC (Mean ± SD in %)	82.6 ± 6.98	83.51 ± 7.14	83 ± 6.63	81.5 ± 7.15
Smoking (Y/N)	37/48	17/12	3/20	18/15
Alcoholism (Y/N)	37/48	17/12	4/19	16/17

(SD: standard deviation, BMI: body mass index, Y: yes, N: No, FVC: forced vital capacity, FEV_1_: forced expiratory volume in 1 sec).

**Table 2:** Identified important deregulated molecules in urine of auto rickshaw drivers (ARD) subjects with respect to office workers (OW).

No	Observed ppm	Metabolite	HMDB ID	VIP SCORE	Fold change (FC:ARD/OW)	P-value
1	1.272	L-Isoleucine	HMDB00172	1.79	5.86	0.001
2	1.466	L-alanine	HMDB00161	1.81	3.39	0.001
3	1.648	2-hydroxy valerate	HMDB01863	1.43	3.67	0.004
4	3.985	Histidine	HMDB00177	1.47	5.87	0.001
5	4.123	Proline	HMDB00162	1.43	3.36	0.003
6	6.591	Cisaconitic acid	HMDB00072	1.41	4.60	0.007
7	6.758	Kynurenine	HMDB00684	1.90	11.17	0.0001
8	6.797	P-cresol sulfate	HMDB11635	1.49	3.70	0.006
9	6.833	4-aminohippuric acid	HMDB01867	1.76	4.20	0.001
10	7.375	Phenylalanine	HMDB00159	1.40	3.37	0.001
11	7.480	Benzoic acid	HMDB01870	1.70	3.31	0.001

VIP: variable importance in projection.
