# Supplementary material for: Biofluid metabotyping of occupationally exposed subjects to air pollution demonstrates high oxidative stress and deregulated amino acid metabolism
Source: Sci Rep. 2016 Oct 21;6:35972. doi: 10.1038/srep35972 (PMC5073280; doi:10.1038/srep35972)
Supplement: Supplementary Information [file srep35972-s1.pdf]

Supplementary Information

Biofluid metabotyping of occupationally exposed subjects to air pollution demonstrates high oxidative stress and deregulated amino acid metabolism.

Surya Narayan Pradhan[1,2], Aleena Das[3], Ramovatar Meena[1], Ranjan Kumar Nanda[3,*], Paulraj Rajamani[1,*]

[1]School of Environmental Sciences, Jawaharlal Nehru University, New Delhi, India

[2]School of Life Sciences, Sambalpur University, Sambalpur, India

[3]Immunology Group, International Centre for Genetic Engineering and Biotechnology (ICGEB), New Delhi, India

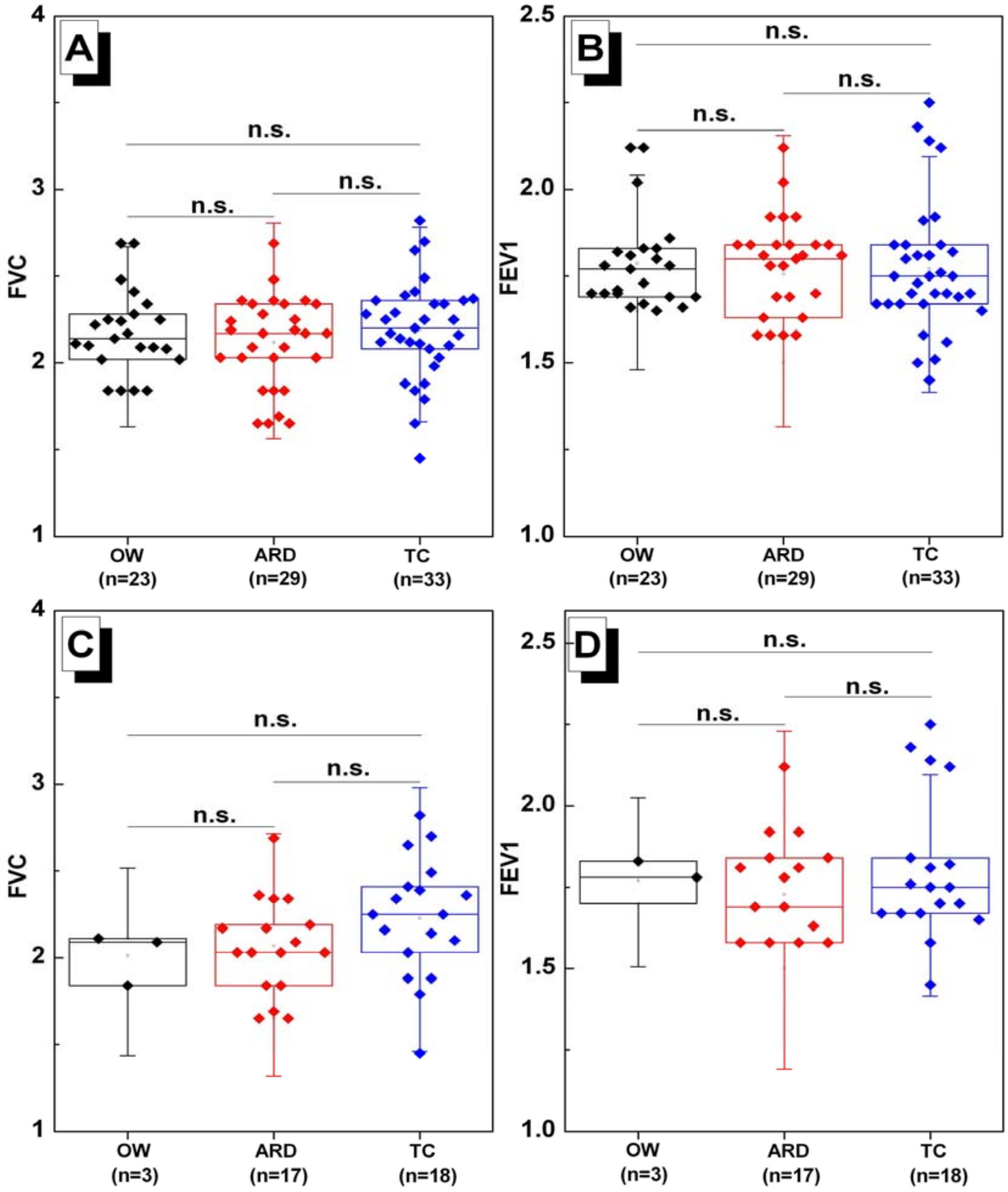

Figure S1: Respiratory parameters of all study subjects belonging to office worker (OW), auto rickshaw driver (ARD), and traffic cop (TC) groups showed similar profile. Box and whisker plot of (A) Forced Vital Capacity (FVC); (B) Forced Expiratory Volume in 1 sec (FEV_1) of OW (n=23), ARD (n=29) and TC (n=33); (C) FVC and (D) FEV_1 of Smokers OW (n=3), ARD (n=17) and TC (n=18) shows insignificant difference between the study groups (n.s.: not significant at 95% confidence of Wilcoxon-Mann–Whitney test. Black diamond shows OW subjects, red for ARD and blue diamond's for TC. Number of subjects used in this study is presented in parenthesis.

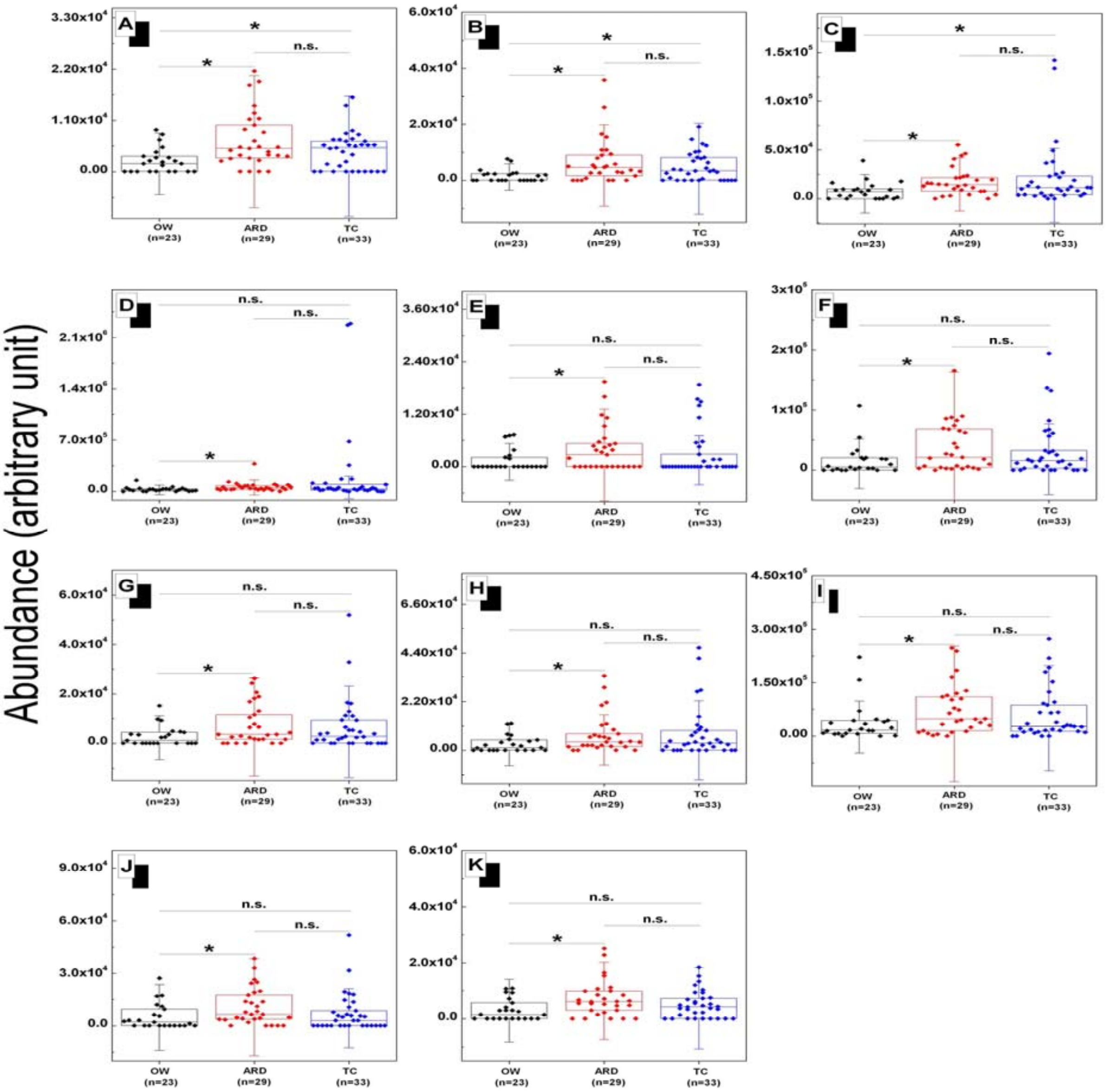

Figure S2: Difference in abundances of individual important metabolites in all study subjects belonging to three study groups (office worker: OW; auto rickshaw driver: ARD; and traffic cop: TC). Box and whisker plots showing difference in abundances of 11 important metabolites (A) 2-hydroxy valerate, (B) Histidine, (C) Cis-aconitic acid, (D) Kynurenine, (E) 4-amino hippuric acid, (F) Phenylalanine, (G) Isoleucine, (H) Alanine, (I) Proline, (J) P-cresol sulfate and (K) Benzoic acid in all three study groups (*: p value < 0.05 of Wilcoxon-Mann–Whitney test and n.s. are not significant at 95% confidence). Black diamond shows OW subjects, red for ARD and blue diamond's for TC. Number of subjects used in this study is presented in parenthesis.

Table S1. Annual (Aug 2013 to July 2014) $PM_{2.5}$ concentration as tabulated from one of the station of the study area.

Table S2. Annual (Aug 2013 to July 2014) PM_{10} concentration as tabulated from one of the station of the study area.
